# Vibration Measurement Method of a String in Transversal Motion by Using a PSD

**DOI:** 10.3390/s17071643

**Published:** 2017-07-17

**Authors:** Che-Hua Yang, Tai-Chieh Wu

**Affiliations:** Graduate Institute of Manufacturing Technology, National Taipei University of Technology, Taipei 10608, Taiwan; chyang@ntut.edu.tw

**Keywords:** position sensitive detector (PSD), dynamic natural frequency measurement, vibration analyze

## Abstract

A position sensitive detector (PSD) is frequently used for the measurement of a one-dimensional position along a line or a two-dimensional position on a plane, but is more often used for measuring static or quasi-static positions. Along with its quick response when measuring short time-spans in the micro-second realm, a PSD is also capable of detecting the dynamic positions of moving objects. In this paper, theoretical modeling and experiments are conducted to explore the frequency characteristics of a vibrating string while moving transversely across a one-dimensional PSD. The theoretical predictions are supported by the experiments. When the string vibrates at its natural frequency while moving transversely, the PSD will detect two frequencies near this natural frequency; one frequency is higher than the natural frequency and the other is lower. Deviations in these two frequencies, which differ from the string’s natural frequency, increase while the speed of motion increases.

## 1. Introduction

The vibration frequencies of structures or components are frequently used for diagnostic or measurement purposes in engineering systems. Slender or thin engineering elements such as belts, cables, chains, ropes and spokes can be referred to as strings because of their shape and harmonic vibration behavior. Over the past few decades, there have been several approaches to developing optical measurements of string vibrations, such as laser interferometers [1,2,3,4], fiber-optical sensors [5,6,7,8], high-speed video cameras [9,10,11,12,13], opto-electronic motion sensors [14,15] and digital image correlation (DIC) [16,17,18]. Using light-emitting and detection sensors, the motion of a vibrated string can easily be captured in order to perform non-contact measurements. These optical techniques have enabled us to make measurements in dangerous environments and conditions while also providing a wide range of detectable frequencies. However, these techniques sometimes have limited applications due to the higher equipment costs of items such as high-speed cameras and laser interferometers, as well as the costs associated with specialized manpower and preparation.

**Figure 1:**
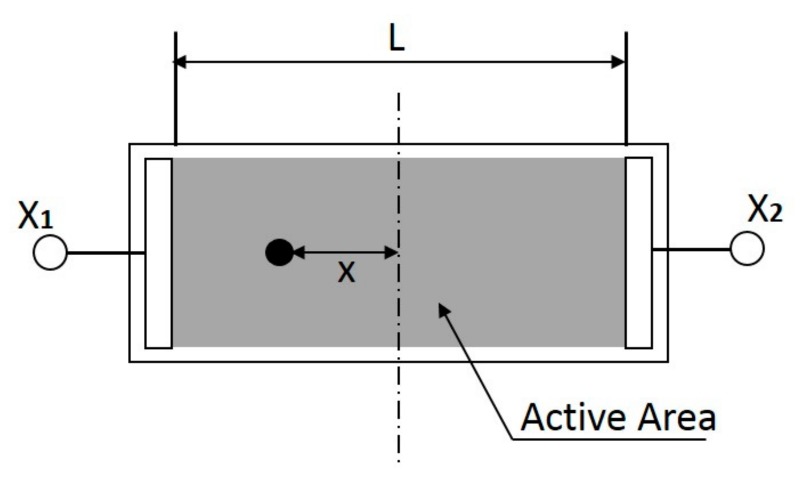
Schematic of a position sensitive detector (PSD).

On the other hand, the position sensitive detector (PSD) has become one of the most important components in this area because it makes good use of lasers in measuring distance, displacement and vibration. A PSD is an optoelectronic position sensor that utilizes photodiode surface resistance with a high accuracy; it also has a fast response speed and a comparatively wide spectrum. As shown in Figure 1, a one-dimensional PSD utilizes a lateral photoelectric effect to produce output signals proportional to the x-coordinates of the centroid of the light spot on the detector [19]. Although the PSD generally detects the movement of a light spot, it can also detect a dark spot on a bright background. The bright background mode is adopted in this study.

There have been extensive studies on PSDs because of their numerous applications in the field of engineering. Devices used for human eye movement monitoring, machine tool alignment, vibration analysis and path tracking all utilize PSDs [20,21,22,23,24,25]. Unlike discrete element detectors such as charge-coupled device (CCD), a PSD can provide continuous position data and features a high position-resolution. At the same time, PSDs have relatively good dynamic reactions, with their response times being in the micro-second realm. Even though they possess verified advantages in terms of their ability for precise positioning and their fast response, studies investigating the use of PSDs to measure string vibration have rarely been done. This is perhaps because the use of PSDs in measuring vibration characteristics is still limited to non-movement or movement at speeds much lower than that of vibration motions [26,27,28]. This research extends the applications of a PSD by characterizing the vibration frequency of a transversely moving string. A theoretical model has been developed for the explanation and prediction of dual-frequency characteristics while a vibrating string transversely moves across a PSD. In addition, a series of measurements were conducted to obtain detailed information regarding a string’s vibration behaviors and also to verify the theory.

## 2. Theoretical Model for a Vibrating String Moving across a PSD

**Figure 2:**
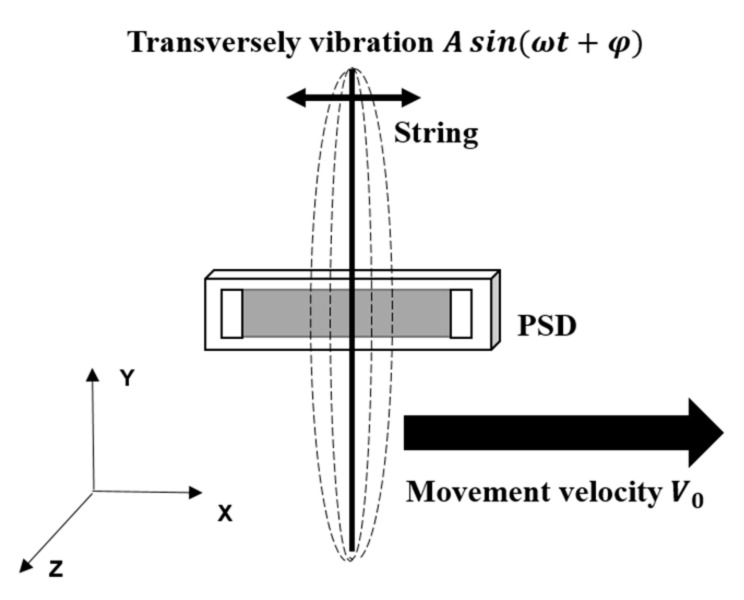
A schematic showing a vibrating string moving across a PSD.

A theoretical model was developed to describe how the PSD would detect the signals of a vibrating string moving in a transverse direction. As shown in Figure 2, the string vibrates with an amplitude of A and frequency of ω. At the same time, the string moves with a constant velocity $\mathrm{of}\;V_{0}$ across the PSD. 

The vibration motion detected by the PSD is considered as a superposition of a harmonic motion due to its vibration together with its transverse motion at a constant velocity. For the harmonic motion, the string’s position x(t) is represented as:

(1) x(t) = A sin(wt+φ).

where A is amplitude, ω is angular frequency and ϕ is phase of the harmonic vibration motion. When the transverse motion at a constant velocity of V_0_ is superimposed, the position of the string can be described as:

(2) $$x\left(t\right)\;=\;V_{o}t\;+\;\mathrm{A sin}\left(\omega t\;+\;\varphi \right)$$

The period, or the inverse of the frequency, of the moving vibrating string can be determined from time intervals where its movement velocities are zero. Thus, a differentiation with respect to time in Equation (2) is considered as:

(3) $$v\left(t\right)\;=\;\frac{\mathrm{dx}(t)}{\mathrm{dt}}{\;=\;V}_{0\;}+\;A\omega \cos(\omega t\;+\;\varphi )\;=\;0,\;\mathrm{or}$$

(4) $$\cos(\omega t\;+\;\varphi )\;=\;\frac{-V_{0}}{A\omega }$$

**Figure 3:**
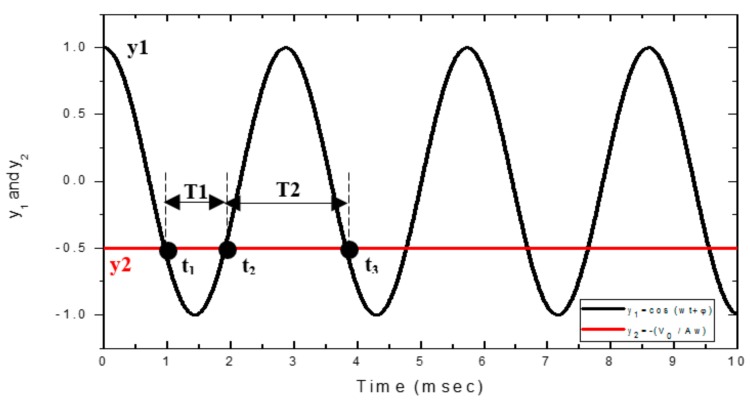
Functions y_1_ and y_2_ and their intersections.

The solution of Equation (4) can be treated as finding the intersections of the two time-dependent functions $y_{1}=\cos(\omega t\;+\;\varphi )$ and $y_{2}\;=\;\frac{-V_{0}}{A\omega }$ The values and their intersections for the functions y_1_ and y_2_ are shown in Figure 3. Here we assume $A=4.5\times 10^{-4}$ m, V_0_ = 0.5 m/s, f_0_ = 349 Hz (T = 1/f_0_ = $2.85\;\times \;10^{-3}$ s) and φ = 0.

The solutions of Equation (4) correspond to the intersections of the two functions y_1_ and y_2_. Since the string vibrates harmonically, this solution pattern will be repeated periodically, as the vibrating string moves across the PSD. As shown in Figure 3 for an arbitrary time interval, the first three solutions are labeled as t_1_, t_2_ and t_3_, which are associated with two vibration periods: T_1_ = t_2_ − t_1_ and T_2_ = t_3_ − t_2_. It can be seen that T_1_ < T and T_2_ > T, where T is the static vibration period with T = 1/(2πf_0_). Hence, for a moving vibrating string with a vibration frequency f_0_ = 1/(2T), a pair of frequencies f_1_ = 1/(2T_1_) and f_2_ = 1/(2T_2_) can be observed from the PSD signal. This dual-frequency phenomenon is similar, but not the same as, the Doppler effect, which describes the shift in frequencies from a moving oscillation source observed from a static position. As opposed to laser Doppler vibrometry (LDV), we do not take the wavelength of the laser into account. Instead, a one-dimensional PSD is used to record the displacement, including the movement path and the transverse vibration of a string. The dual-frequency phenomenon is not only caused by the movement velocity but is also influenced by the vibration of the string itself.

**Figure 4:**
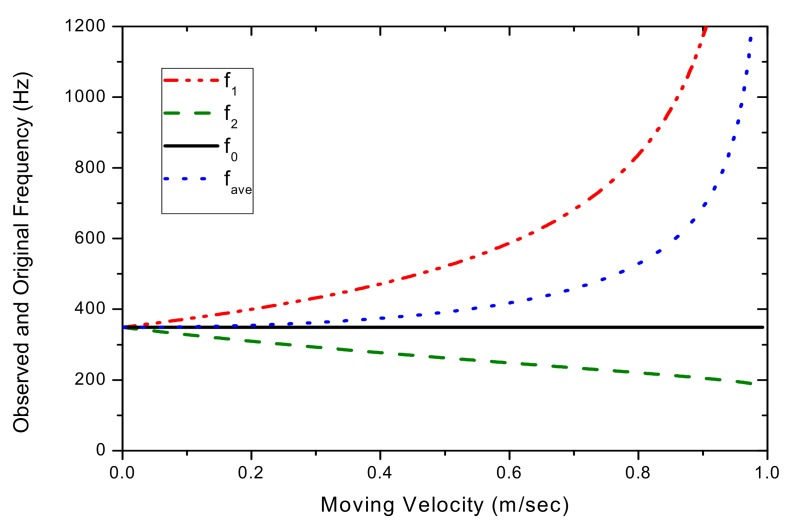
Shifted frequencies f_1_ and f_2_ and their averages compared with f_0_.

**Figure 5:**
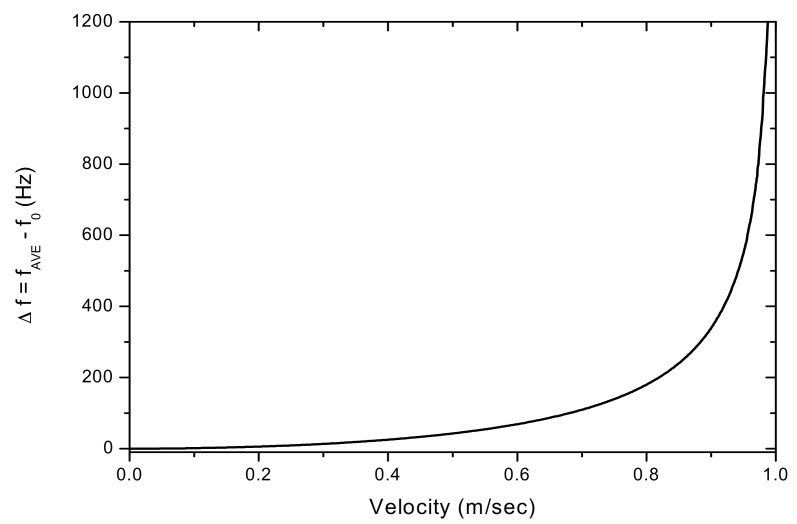
Δf as a function of the string’s movement velocity.

After solving Equation (4), Figure 4 shows the shifted frequencies f_1_ and f_2_ measured by the PSD and as a function of the transverse movement velocity of the string. Figure 4 also shows the string’s original vibration frequency (f_0_). It can be seen that f_1_ has a more pronounced frequency-shift effect than that of f_2_ as the string’s movement velocity increases. With the observed frequencies shifted in opposite directions, one may assume to be able to use the average f_ave_ = (f_1_ + f_2_)/2 as an approximation for the original frequency f_0_. In Figure 4, the averaged frequency is also plotted for comparison with f_0_. It demonstrates that the difference between f_ave_ and f_0_ is substantial. Figure 5 shows $\Delta {f\;=\;f}_{\mathrm{ave}}{\;-\;f}_{0}$ and the deviation of f_ave_ from f_0_, as a function of the string’s movement velocity. The difference between f_ave_ and f_0_ is 1.6% for V_0_ = 0.2 m/s, 7.2% for 0.4 m/s, 19.7% for 0.6 m/s and 51.5% for 0.8 m/s, respectively.

**Figure 6:**
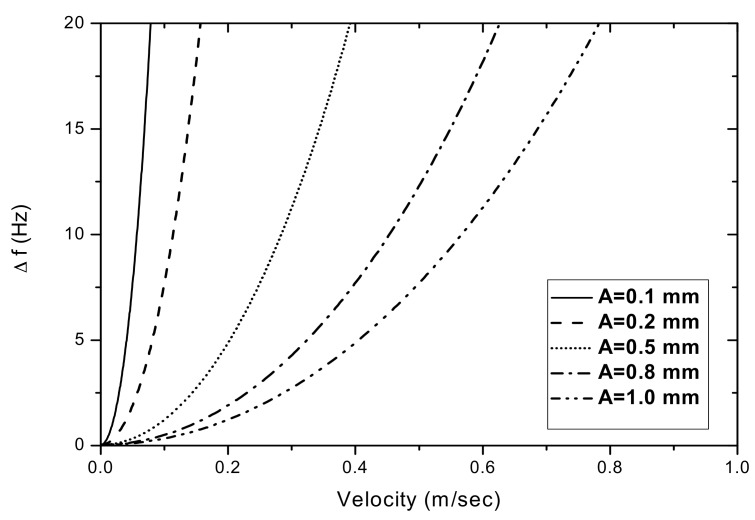
Δf as a function of V_0_ for various vibration amplitudes (A).

As hinted in Equation (4), another parameter influencing Δf is the vibration amplitude (A). Figure 6 shows the Δf as a function of the movement velocity (V_0_) for various vibration amplitudes. It shows that the frequency shifting is due to the string’s movement and is more pronounced for vibrations with smaller amplitudes.

## 3. Experiment

**Figure 7:**
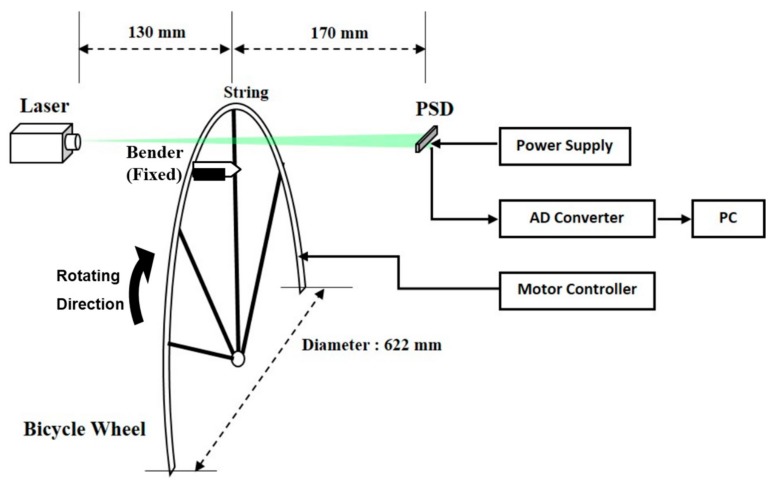
A schematic of the experimental setup.

Experiments to observe the PSD-detection of vibrations from a vibrating string stretched on a rotating bicycle wheel were conducted. The experiment setup is shown in Figure 7. A 100 mW line-expanded laser module (OP Mount Instrument Inc., Taoyuan, Taiwan) with a wavelength of 532 nm was utilized as a light source. A one-dimensional PSD (HAMAMATSU S3932, Hamamatsu, Japan) with an active area of 1 × 12 mm and a spectral range covering 320 nm to 1100 nm was used to detect the shadow cast by the vibrating string. A signal processing circuit board (HAMAMATSU C3683-02) provided power to the PSD and amplified the signal. The rise time for this PSD system was 3 μs and the calibration for the PSD was 10 mm/V. An analog-to-digital converter (DAQ6351, National Instrument, Austin, TX, USA), together with a personal computer, was used to acquire data from the vibration signals for further analysis. For each vibration signal waveform, a total of 8000 samples were acquired, with a sampling rate of 40 kHz.

The 28 strings were assembled onto a bicycle wheel, but only one string was used for the natural frequency measurement, in order to satisfy the demands of repeatability and reproducibility. The diameter of the wheel was 622 mm, excluding the tire. The string was made of stainless steel, with a length of 260 mm and a diameter of 1.82 mm (SAPIM Co., Antwerpen, Belgium). Here, the string’s movement can be regarded as a linear path, because the width of the PSD is only 12 mm. The detected region is relatively small when compared with the rotating motion of the entire wheel; for this reason, we consider the string as running on a linear path. The distance between the laser and the string was 130 mm, with a distance of 170 mm from the string to the PSD. The wheel was driven by a servo-motor for rotational speed control, as listed in Table 1. An adjustable, flexible plastic bender was used to trigger the vibration at its fundamental natural frequency as the string passed on the rotating wheel. An independent measurement for the natural frequency of the string was performed with a frequency meter (Type 6, CLAVIS Co., Tyne and Wear, UK) for comparison with the PSD.

This sensor system has a limitation of its maximum frequency. The maximum frequency for the proposed method will be limited by two factors: the PSD frequency response and the speed of the ADC (analog-to-digital conversion). With the current system configuration, the PSD has a rise time of 3 μs and the ADC can sample up to 1.25 MS/s. This configuration can determine the motions of the string up to at least 10 kHz. String motion at a higher frequency can be measured by updating the instrumentation, but is not limited by the measurement principle. Since this method is an FFT-based frequency determining method, the frequency accuracy will naturally be limited by the samples of FFT. With zero-peddling, the number of samples was extended to 80,000. With a sampling rate of 80 KHz, the limitation of the frequency resolution will be 1 Hz.

## 4. Results and Discussions

**Figure 8:**
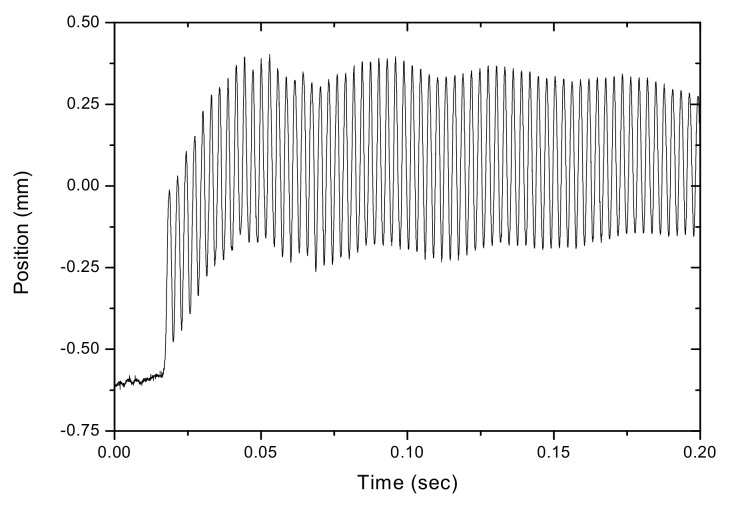
Vibration signal for the non-moving string, detected with the PSD.

**Figure 9:**
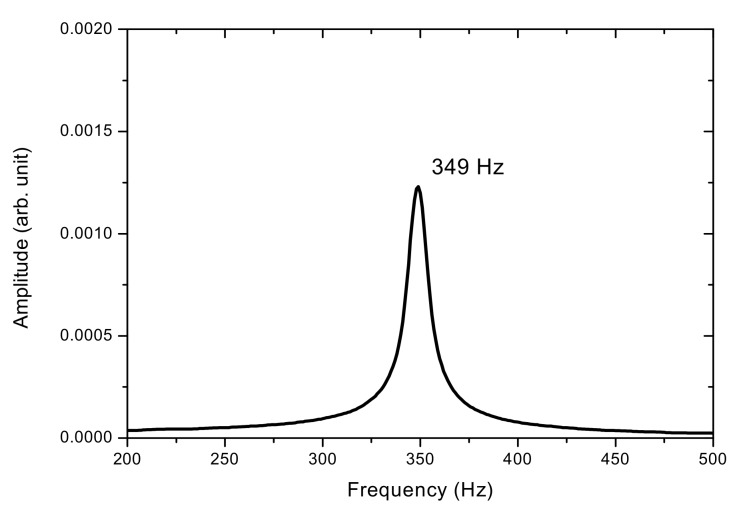
Power spectrum density for the non-moving vibrating string.

The results of the measured frequency characteristics for a non-moving vibrating string are presented first. While the wheel was not moving, the vibration frequency of the stretched string was measured with the PSD system and the independent frequency meter. For the observed string, the frequency meter showed a frequency reading of 349 Hz. On the other hand, Figure 8 shows the acquired vibration signal detected by the PSD. The average vibration amplitude (A) for this motion was 0.51 mm. Figure 9 shows the frequency spectrum after a fast Fourier transform (FFT) of the acquired signal in Figure 8. For the FFT, zero-padding was used to achieve a frequency resolution of up to 1 Hz. As shown in Figure 9, a single natural frequency was measured by the PSD at 349 Hz, which is on a par with the frequency meter measurement. At this point, a frequency measurement system using PSD can be considered to be a trustworthy system when measuring non-moving string vibration frequency.

**Figure 10:**
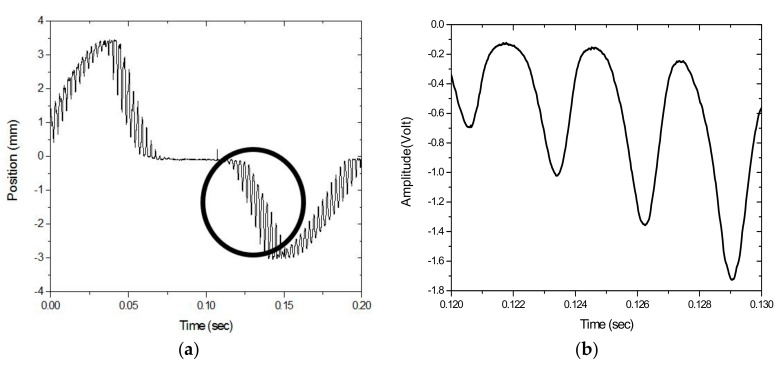
(**a**) PSD-detected signal and (**b**) the enlarged portion of the motion of the vibrating string at $3.9\;\times \;10^{-2}$ m/s.

**Figure 11:**
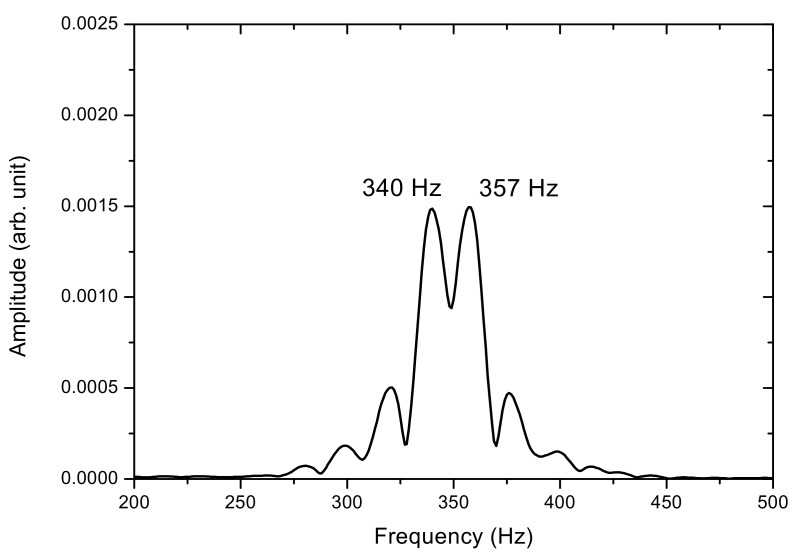
Frequency spectrum of the motion of the vibrating string at $3.9\;\times \;10^{-2}$ m/s.

The measurements taken for the frequency characteristics of the vibrating string as the wheel is rotated from the PSD are here investigated. For string motion at $3.9\times 10^{-2}$ m/s (or 39 mm/s) while the wheel rotates at 2.00 rpm, Figure 10a shows the PSD-detected signal; a portion is enlarged in Figure 10b. In contrast with the waveform in Figure 8, which shows string vibration without transverse motion, the signal in Figure 10a shows the vibration of the string when measured simultaneously as it moves across the PSD. Two types of motions contribute to the PSD-detected signal; one is the vibration, while the other is the transverse motion. The vibrational signal is shown as a higher-frequency carrier, which is modulated by a lower-frequency envelope when the string enters and exits the PSD’s 1 × 12 mm active area. As shown in Figure 10b, the combined PSD-detected movement and vibration motion is not a sinusoidal waveform. The PSD-detected signal deviates from a perfect sinusoidal pattern, with its rising parts slightly steeper than the falling ones, hinting at a dual-frequency nature in its frequency spectrum. Indeed, the frequency spectrum of the vibration signal, shown in Figure 11, has two peaks; one at f_1_ = 357 Hz and another at f_2_ = 340 Hz; compared to that of a string vibrating without transverse motion, which has a frequency of f_0_ = 349 Hz, where f_1_ has a frequency shift of +8 Hz and f_2_ of −9 Hz. Theoretical calculations by using Equation (4) predict frequency shifts of f_1_ = +8.9 Hz and f_2_ = −8.4 Hz; these predictions agree well with the measurements and are accurate to within ±1 Hz.

**Figure 12:**
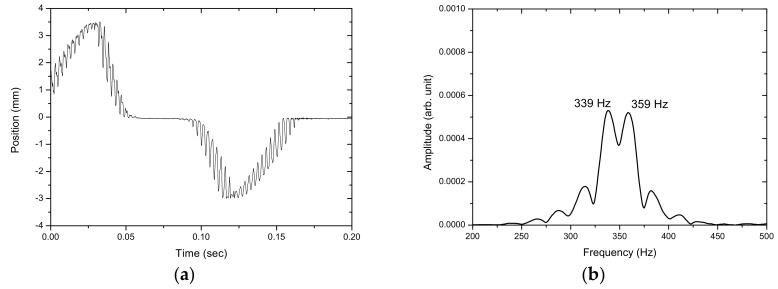
(**a**) The PSD-detected signal and (**b**) the frequency spectrum of the motion of the vibrating string at $4.8\;\times \;10^{-2}$ m/s.

**Figure 13:**
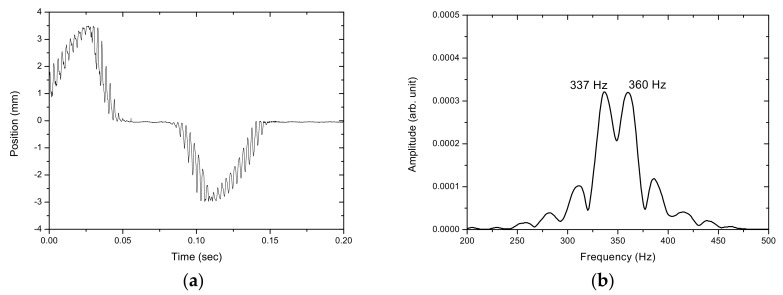
(**a**) The PSD-detected signal and (**b**) the frequency spectrum of the motion of the vibrating string at $5.4\;\times \;10^{-2}$ m/s.

**Figure 14:**
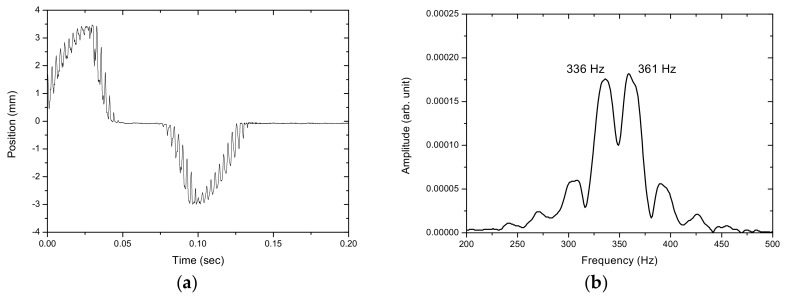
(**a**) The PSD-detected signal and (**b**) the frequency spectrum of the motion of the vibrating string at $5.7\;\times \;10^{-2}$ m/s.

**Figure 15:**
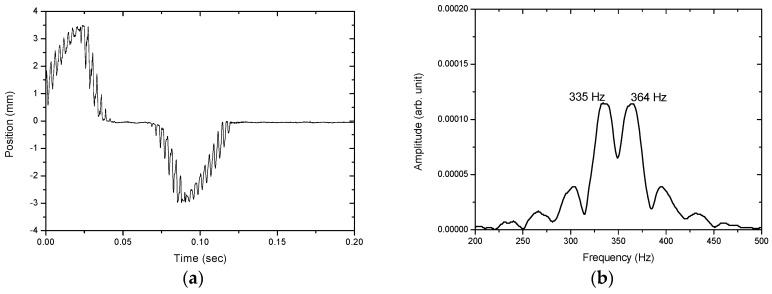
(**a**) The PSD-detected signal and (**b**) the frequency spectrum of the motion of the vibrating string at $6.3\;\times \;10^{-2}$ m/s.

**Figure 16:**
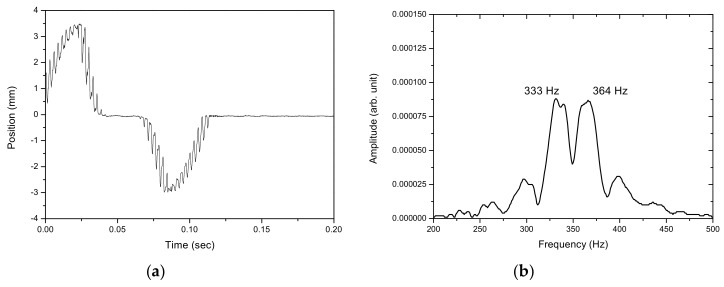
(**a**) The PSD-detected signal and (**b**) the frequency spectrum of the motion of the vibrating string at $6.8\;\times \;10^{-2}$ m/s.

**Figure 17:**
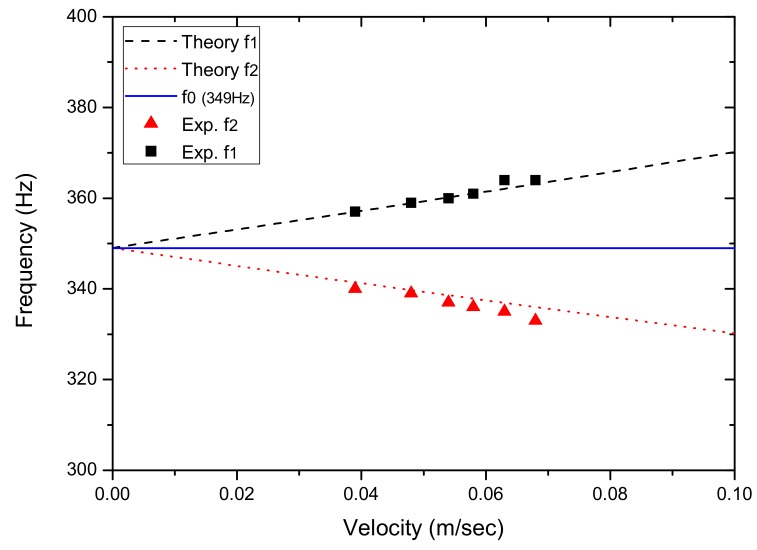
Predicted and measured frequencies at various movement velocities.

**Figure 18:**
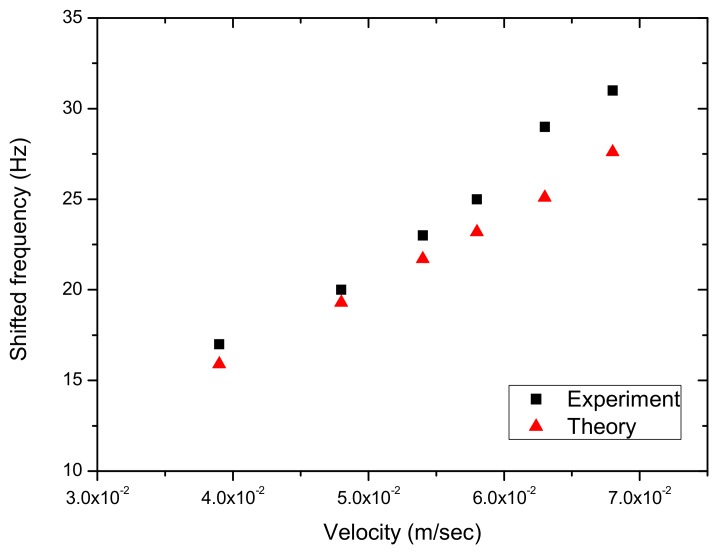
A comparison of the measured and predicted shifted frequencies.

Figure 12, Figure 13, Figure 14, Figure 15 and Figure 16 show the PSD-detected time-domain signals and frequency spectra of the vibrating string when moving at higher speeds of $4.8\;\times \;10^{-2}$, $5.4\;\times \;10^{-2}$, $5.7\;\times \;10^{-2}$, $6.3\;\times \;10^{-2}$ and $6.8\;\times \;10^{-2}$ m/s, respectively. As shown in Table 2, compared to f_1_ − f_2_ = 17 Hz in the previous case, where V_0_ = $3.9\;\times \;10^{-2}$ m/s, it is 20 Hz, 23 Hz, 25 Hz and 29 Hz for these cases with higher movement velocities. Figure 17 shows the measured frequencies as a function of movement velocity compared with the theoretical predictions. The measurement results are in line with the theoretical predictions. A comparison of the PSD-detections and the predictions of the shifted frequencies are listed in Table 3 and shown in Figure 18. Higher discrepancies can be seen when the movement velocity is higher than 0.06 m/s. The errors can be attributed to unclear peaks in the spectra, such as those shown in Figure 14b and Figure 16b. These motions may contain harmonic vibrations, but also may have inherited influences from the bender when triggering the vibration. This error may be reduced by delaying the signals until a certain time after the initial trigger from the bender. Another cause that may lead to the unclear peaks could be an insufficient time to acquire the vibration data of the moving string. The active area of the PSD is only 1 × 12 mm, giving an acquisition time of less than 0.15 s as the moving speed of the string increased. The number of samples and the sampling rate are inadequate to demonstrate the full behavior of string vibration. 

## 5. Conclusions

A position sensitive detector (PSD) is well-known for its applications in the detection of positions and the vibrational characteristics of objects in static or quasi-static conditions. This research extends the application capabilities of a PSD and enables the characterization of the vibration frequency from transversely moving string. Dual-frequency spectra are found to be associated with the vibration of a moving string, with one of the frequencies being higher than the original vibration and the other being lower. The average of the two PSD-detected frequencies cannot represent the original vibration frequency of the string; instead, the averaged frequency is always higher, with a discrepancy which increases as the movement velocity increases. The measured frequencies and frequency-shifting due to the moving velocity of a string agree well with the theoretical predictions. With the natural frequencies of a moving string able to be reliably measured, further important properties such as the string tension can be obtained. This study proposes the use of a one-dimensional PSD for measuring the natural frequencies of moving strings with sufficient accuracy in a cost-effective way.

## Figures and Tables

**Table 1:** Wheel rotation and the linear velocities of the string.

Rotation Speed (rpm)	Linear Movement Velocity (m/s)
2.00	$3.9\;\times \;10^{-2}$
2.40	$4.8\;\times \;10^{-2}$
2.73	$5.4\;\times \;10^{-2}$
2.96	$5.8\;\times \;10^{-2}$
3.21	$6.3\;\times \;10^{-2}$
3.43	$6.8\;\times \;10^{-2}$

**Table 2:** Frequencies measured with the PSD at various wheel rotation speeds.

Wheel Rotation Speed (m/s)	Measured Frequencies (f_1_, f_2_,Hz)	f_2_ − f_1_ (Hz)
$3.9\;\times \;10^{-2}$	340, 357	17
$4.8\;\times \;10^{-2}$	339, 359	20
$5.4\;\times \;10^{-2}$	337, 360	23
$5.7\;\times \;10^{-2}$	336, 361	25
$6.3\;\times \;10^{-2}$	335, 364	29
$6.8\;\times \;10^{-2}$	333, 364	31

**Table 3:** Differences between measured and theoretical shifted frequencies at various velocities.

String Linear Velocity (m/s)	Interval of Measured Shifted Frequencies f_1_, f_2_	Interval of Theoretical Shifted Frequencies f_1_, f_2_	Difference
$3.9\;\times \;10^{-2}$	17	15.9	6%
$4.8\;\times \;10^{-2}$	20	19.3	3%
$5.4\;\times \;10^{-2}$	23	21.7	6%
$5.8\;\times \;10^{-2}$	25	23.2	7%
$6.3\;\times \;10^{-2}$	29	25.1	13%
$6.8\;\times \;10^{-2}$	31	27.6	11%
